# Deoxyelephantopin and Its Isomer Isodeoxyelephantopin: Anti-Cancer Natural Products with Multiple Modes of Action

**DOI:** 10.3390/molecules27072086

**Published:** 2022-03-24

**Authors:** Tahir Mehmood, Chatchai Muanprasat

**Affiliations:** Chakri Naruebodindra Medical Institute, Faculty of Medicine Ramathibodi Hospital, Mahidol University, Bang Pla, Bang Phli, Samut Prakan 10540, Thailand; tahir_nanotech@yahoo.com

**Keywords:** deoxyelephantopin, isodeoxyelephantopin, *Elephantopus scaber*, sesquiterpene lactone, apoptosis, cancer

## Abstract

Cancer is a leading cause of morbidity and mortality worldwide. The development of cancer involves aberrations in multiple pathways, representing promising targets for anti-cancer drug discovery. Natural products are regarded as a rich source for developing anti-cancer therapies due to their unique structures and favorable pharmacology and toxicology profiles. Deoxyelephantopin and isodeoxyelephantopin, sesquiterpene lactone compounds, are major components of *Elephantopus scaber* and *Elephantopus carolinianus*, which have long been used as traditional medicines to treat multiple ailments, including liver diseases, diabetes, bronchitis, fever, diarrhea, dysentery, cancer, renal disorders, and inflammation-associated diseases. Recently, deoxyelephantopin and isodeoxyelephantopin have been extensively explored for their anti-cancer activities. This review summarizes and discusses the anti-cancer activities of deoxyelephantopin and isodeoxyelephantopin, with an emphasis on their modes of action and molecular targets. Both compounds disrupt several processes involved in cancer progression by targeting multiple signaling pathways deregulated in cancers, including cell cycle and proliferation, cell survival, autophagy, and invasion pathways. Future directions of research on these two compounds towards anti-cancer drug development are discussed.

## 1. Introduction

Cancer is a leading cause of mortality in both developed and developing countries, and remains a major threat to public health worldwide [1]. According to the World Health Organization (WHO), before the age of 70 years, cancer is the first or second leading cause of mortality in 112 of 183 countries, and the third or fourth leading cause of mortality in 23 countries [2]. WHO statistical estimation shows that there will be 27 million new cancer cases and 17.5 million cancer-related deaths by 2050 [3]. According to the International Agency for Research on Cancer (IARC), approximately 19.3 million new cancer cases and almost 10 millions of cancer deaths occurred in 2020, excluding comorbidities related to COVID-19 deaths [2]. In Europe and the United States, 2.6 million cancer cases are reported each year [4]. In fact, numbers of cancer cases and deaths are expected to grow rapidly due to population aging and lifestyle behaviors that increase cancer risk. In addition, accumulative scientific evidence suggests that cancer is caused by the deregulation of various essential genes encoding for signaling proteins, including anti-apoptotic proteins, transcription factors, tumor suppressers, inhibitors of apoptosis, growth factors, and growth factor receptors [4,5]. Since cancer is not a single step process and develops through multiple aberrations, developing anti-cancer drugs targeting multiple targets is considered a promising therapeutic approach. Much attention has been paid to the investigation of the anti-cancer activities of natural products, especially originating from plants, as phytochemicals are likely to have a good safety profile and target multiple mechanisms central to cancer development.

**Figure 1:**
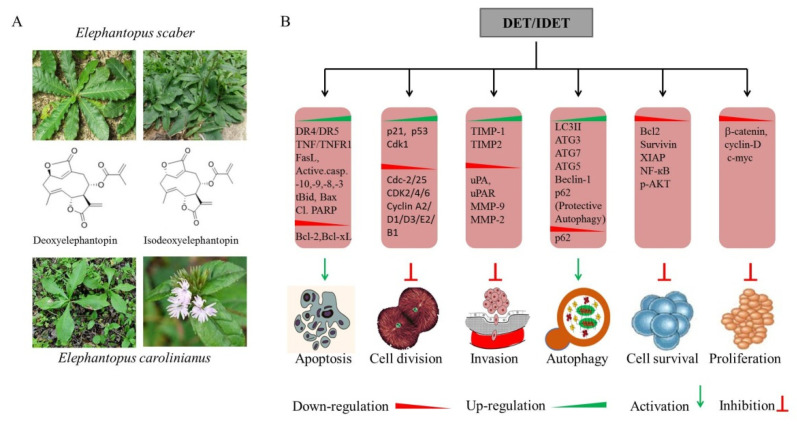
Deoxyelephantopin (DET) and isodeoxyelephantopin (IDET) (**A**): Chemical structure and natural sources of DET and IDET. (**B**): DET/IDET inhibits cancer progression and development by inhibiting cancer cell proliferation, survival, invasion, and cell cycle progression, and ultimately induces apoptosis and autophagy by modulating the expression of various genes.

Phytochemicals have extensively been used in traditional medicine to treat a wide spectrum of diseases. Plants, as an important component of many traditional medicines, provide the richest source of small molecules with outstanding chemical diversity. Their value can be appreciated by the fact that 87% of human ailments, including cancer, inflammations, and bacterial and parasitic infections, are being be treated with plant-derived formulations [4]. The WHO reports that 80% of global populations still rely on plant-derived formulations for their primary health care [1,4,6,7]. Statistical data show that natural sources, including plants, marine organisms, and micro-organisms, contribute to more than 60% of commercially available anti-cancer drugs, further highlighting the significance of natural products [8,9]. Furthermore, 200,000 cancer-related deaths and more than 20% of cancer incidence can be prevented through the consumption of fruits and vegetables [1,10,11]. The aim of this review is to summarize and discuss the natural sources, anti-cancer activities, mechanisms of action, and molecular targets of deoxyelephantopin (DET) and its isomeric form, isodeoxyelephantopin (IDET), both of which are sesquiterpene lactone (SL) compounds. The chemical structures and natural sources of DET and IDET are shown in Figure 1A. DET and IDET show cytotoxic activity against several human cancer cell lines by targeting multiple signaling pathways central to cancer development and propagation, as summarized in Figure 1B.

## 2. Natural Sources and Anti-Cancer Activities of DET and IDET

DET and IDET are major components of *Elephantopus scaber Linn* and *Elephantopus carolinianus*, which belong to the family Compositae (Asteraceae), the largest angiosperm family [12,13,14,15]. Sesquiterpene lactones (SLs) are taxonomic markers of the family Asteraceae. *Elephantopus scaber* L., a medicinal plant, is commonly known as “tawmonlar” in Myanmar traditional medicine, “Tutup bumi” in Malaysian, “Elephant’s foot” in English, “Didancao” in traditional Chinese medicines (TCM), and “Gobhi” in Hindi [15,16,17,18,19,20,21,22]. *Elephantopus* genus is widely distributed all over the world, especially in east Asia, southeast Asia, Africa, Australia, India, Europe, and South America [16,21,22,23,24,25]. Only *Elephantopus scaber,* one of the 32 species of genus, is known to grow in India [26,27]. DET and IDET show various biological activities, including anti-bacterial, anti-malarial, anti-diabetic, anti-inflammatory, wound healing, hepatoprotective, and anti-cancer activities [28,29,30]. SLs are considered to be chemotaxonomic markers of the family Asteraceae. Biological activities of the genus *Elephantopus* are mainly attributed to SLs [21]. DET and IDET are main SL components of *Elephantopus scaber* and *Elephantopus carolinianus* [14,22]. Several studies have revealed that DET and IDET are promising compounds with anti-cancer activities against several types of cancer. DET possesses anti-cancer activities against cervical carcinoma [30], and nasopharyngeal carcinoma [31,32], as well as breast [16,28,33], colon [28,34,35], lung [28,33], and liver cancers [36] by targeting several pathways. IDET also shows anti-cancer activities against breast cancer, leukemia, and lung cancer by targeting multiple signaling cascades [17]. Moreover, several studies have shown the safety profile of DET. DET was tested against peripheral blood lymphocytes isolated from healthy individuals. Interestingly, DET at higher concentrations (35 µg/mL) did not induce toxicity in peripheral blood lymphocytes, suggesting that DET-induced toxicity is specific to cancer cells [37]. DET (21.69 ± 0.92 µg/mL; 72 h) showed relatively little cytotoxicity against CCD841CoN normal colon cells when compared to HCT116 cells (0.73 ± 0.01; 72 h), revealing a 30-fold difference in cytotoxicity [34]. Likewise, IDET had good safety profile to normal lymphocytes, and showed cancer-specific cytotoxicity against T47D breast cancer cells and A549 cancer cells [16]. The selective cytotoxicity of both DET and IDET against cancer cells suggests that both compounds selectively target pathways in cancer cells.

## 3. Effect of DET and IDET on Apoptosis Pathways

Apoptosis, or programmed cell death, is regulated by a series of intracellular interlinked cell signaling events to remove unwanted and cancer cells [38,39]. It is characterized by specific phenotypic and biochemical hallmarks. Phenotypic hallmarks of apoptosis include cell shrinkage, membrane blebbing, pyknosis (chromatin condensation), karyorrhexis (nuclear fragmentation), and apoptotic body formation containing cytoplasmic and nuclear material of deceased cells [28,39]. Biochemical hallmarks of apoptosis include caspase-3 activation [40,41,42,43]. There is equilibrium between cell proliferation and apoptosis. Old or unwanted cells are replaced by new cells. Disruption of this equilibrium can lead to the development of pathological conditions/processes, including tumors, auto-immunity, neurodegeneration, and developmental abnormalities [44,45,46]. The malfunction of apoptosis regulators can reduce apoptosis and promote tumorigenesis. Generally, natural compounds affect apoptosis regulators to induce apoptosis-mediated cell death in various cancers [47,48]. Several studies have reported that DET and IDET induce apoptosis in various types of cancer cells through multiple mechanisms, such as cell cycle arrest induction, the promotion of mitochondrial dysfunction, reactive oxygen species (ROS) induction, Bcl-2 family protein modulation, the inhibition of nuclear factor-kappa B (NF-κB), and the inhibition of signal transducers and activators of transcription 3 (STAT3) activation [28,29,30,31,32,33,34,35,36,37].

### 3.1. Cell Cycle Arrest-Mediated Apoptosis

A fractional increase in cells at a particular checkpoint of the cell cycle is one of the major causes of cell death in tumor cells. Precise division of cells is guaranteed by a synchronized sequence of events, regulated by organized interactions of several cyclins with their respective cyclin-dependent kinases (CDKs). At various checkpoints, each phase of cell cycle is completed before moving to the next phase. It is well established that clinically used drugs induce apoptosis through cell cycle arrest at various checkpoints, especially at the G1/S phase and G2/M phase. p53, a tumor suppresser gene, is a key regulator of cell cycle progression and mediator of apoptosis. It activates its downstream effectors, including p21, and Bcl-2-like protein 4 (Bax), which eventually leads to cell cycle arrest, DNA repair, and apoptosis. The negative regulation of CDKs by cyclin-dependent kinase inhibitors (CDKIs), including p21_Waf1/Cip1_ and p27_Cip/Kip_, plays important roles in cell cycle arrest [49,50,51,52]. The p21 and p27 proteins are cell cycle inhibitors responsible for tumor suppression by inhibiting the activation and binding of cyclins and CDKs in the cancer cell cycle and mitosis [52,53]. The expression of p21 and p27 has been explored in many reports to study their significance in cell cycle arrest during chemotherapeutic drug development. In all eukaryotic cells, for the advancement of G2 to M phase, cyclin B/cdc2 complex is required [54]. p21 inactivates the cyclin B1/cdc2 complex, and induces cell cycle arrest [55]. Several studies have demonstrated that natural compounds, such as taxol and vinca alkaloids, induce cell cycle arrest by targeting microtubules at G2/M phase [55,56,57]. Other anti-cancer agents, including resveratrol and cisplatin, also induce S phase cell cycle arrest in cancer cells [58,59,60].

It has been shown that DET limits the growth of cancer cells at different phases of cell cycle in different cancer cell lines. In uterine leiomyoma (UL) cells, DET (5, 10, and 25 µM) induced cell cycle arrest at the G2/M phase of the cell cycle in a concentration-dependent manner. DET (25 µM) significantly increased DNA content at the G2/M phase (36.61 ± 1.3%) compared to the control (19.88 ± 0.8%). Moreover, DET downregulated cyclin B1 and PCNA mRNA expression in UL cells [61]. DET (0.6–11.6 µM) induced sub-G1 population cell cycle arrest in human prostate carcinoma PC-3 cells, human nasopharyngeal carcinoma CNE cells, and human acute promyelocytic leukemia HL-60 cells [62]. Alteration in the expression levels of various cell cycle regulatory proteins was also observed in DET-induced CNE cell death. The DNA content at the S phase was found to be increased, while the DNA content was increased at the G2/M phase at the concentration of 11.6 µM, but declined at the concentration of 46.5 µM. DET induced S phase arrest by downregulating CDK4/6 and cyclin-D1 and -D3, which are the major regulators of G1/S-phase arrest. Regulators, including cdc2, cdc25, and cyclin B1, control the transition of G2 to the M phase of cell cycle. DET (46.5 µM) induced G2/M phase arrest by downregulating the expression of cdc2, cdc25, and cyclin B1 in CNE cells [32]. It has also been found that DET (2 µg/mL) treatment increased G2/M phase DNA content, while long-term DET treatment (36–48 h) increased sub G1 DNA content in cell cycle arrest analysis in TS/A cells. DET modulated expression of cyclins-A, -D1, and -E, and CDKs-2/4 at an early phase (6–12 h), and increased the ratio of phosphorylated Rb to Rb protein at a later phase (12–48 h) in TS/A cells. CDK1 and cyclin-B1 are the gateways to controlling G2/M phase arrest. However, DET increased inactive phospho-(Tyr15)-CDK1 and decreased cyclin B1 expression from 6 to 24 h in TS/A cells. The expression level of p21 was upregulated by DET in TS/A cells [33]. DET induced G2/M phase arrest in HeLa cells. The DNA content in the G1 phase was found to be decreased by DET (10 and 20 µM), and a population of cells at the G2/M phase was increased by DET (20 µM) in HeLa cells [63]. DET (12.287 µg/mL) induced G2/M phase arrest in A549 cells, with the percentage of cells at the G2/M phase being increased from 5.3% (control) to 39.2%, and those in the G1 phase being decreased from 87.8% (control) to 48.3% [37]. In SiHa cells, DET (4.14 μg/mL; 48 h) induced G2/M phase arrest by downregulating cyclin B1 and cdc2, and upregulating p21 and p53 [30]. Moreover, DET inhibited cell cycle regulators synergistically with cisplatin, resulting in apoptosis in murine melanoma B16 cells. DET (5 µM) alone and a combined treatment of DET (3 µM) and cisplatin (40 µM) decreased G0/G1 phase DNA content and increased G2/M phase DNA content, as compared to the vehicle-treated (control) group by downregulating cyclin A, B1, and D1, along with CDK1 at 12 to 24 h. In B16 cells, DET alone and DET combined with cisplatin altered the p-p53/p53 ratio, and increased p21 at 6 to 24 h, while the p27 level was initially decreased at 6 to 12 h, and then increased at 24 h post-treatment. These findings suggest that DET potentiates the apoptotic effect of cisplatin by inducing both p53-dependent and -independent cell cycle arrest and apoptosis in B16 cells [52].

**Figure 2:**
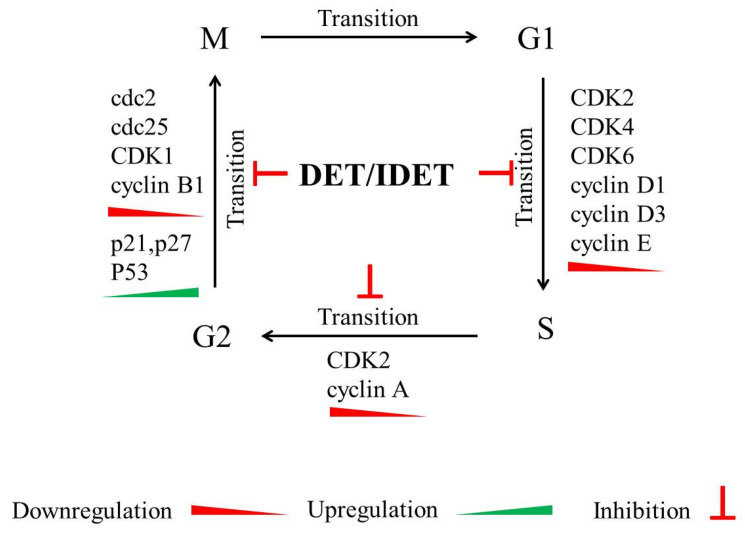
A schematic model of DET- and IDET-induced cell cycle arrest at multiple phases. DET and IDET inhibit S phase transition by downregulating the expression of cyclin A, cyclin D1, cyclin D3, cyclin E, CDK2, CDK4, and CDK6. DET and IDET inhibit G2/M phase transition by downregulating the expression of cdc2, cdc25, cyclin B1, and CDK1 while upregulating the expression of p21_Waf1/Cip1_ and p27_Cip/Kip_ (CDK inhibitors) and p53, a tumor suppresser gene which regulates cell cycle arrest at different phases by inducing p21.

In HCT 116 (colorectal), K562 (chronic myeloid leukemia), KB (oral), and T47D (breast) cancer cells lines, it has been shown that 48 h of DET treatment (7.46, 4.02, 3.35, and 1.86 µg/mL, respectively), increased the G2/M phase DNA proportion with a concomitant decrease in G1 and S phase DNA content, indicating a G2/M phase arrest by increasing p21 expression and decreasing cyclin B1 and cdc2 expression. DET induced p53 expression in HCT 116 and KB cells, indicating that DET induced a p53-dependent cell cycle arrest. In T47D cells carrying a mutant nonfunctional p53, DET increased the expression of mutant p53, and induced cell cycle arrest. Moreover, in K562 cells, where the p53 gene was deleted, no p53 expression was detected after DET treatment; DET also increased p21 expression in K562 cells, indicating that DET induced p53-independent G2/M phase arrest [64]. DET (1.5 and 3 µg/mL) induced S phase arrest in HCT116 cells through the increased phosphorylation in p53 and the upregulation of p21 in a concentration-dependent manner. The increased expression in phosphor-p53 and p21 resulted in the downregulation of CDK2/4 and cyclin A2, B1, D1, and E2 protein expression, all of which are essential for G1 to S phase transition [34]. IDET inhibits G2/M phase transition, and induces G2/M phase arrest. In T47D cells, IDET (1.3 μg/mL) significantly increased the DNA content in the G2/M phase, and induced caspase-3-mediated apoptosis. In A549 cells, IDET (10.46 μg/mL; 48 h) induced cell cycle arrest at the G2/M phase [16]. It has been shown that IDET (4–12 µM) induced G2/M phase cell cycle arrest in CNE1 and SUNE1 cells in a time- and concentration-dependent manner. The effect of IDET appears to be cell-type specific, as it does not induce cell cycle arrest in NP69 normal nasopharyngeal cells [31]. IDET (10 and 25 µM) induced cell cycle arrest in MDA-MB-231 cells at the sub-G1 and G2/M phases. IDET (25 µM) increased the DNA content at the sub-G1 phase and G2/M phase by 3.5-fold and two-folds, respectively. IDET (2.5 and 5 µM) downregulated the mRNA expression of cyclin D1, leading to increased DNA content at the sub-G1 phase in MDA-MB-231 cells [23]. Based on available data, it appears that DET and IDET hold the potential to induce cell cycle arrest-mediated apoptosis at different phases of the cell cycle, including the sub-G1, G1/S, and G2/M phases. DET and IDET limit the growth of cancer cells at different phases of the cell cycle in different cancer cell lines by downregulating CDK1, 2, 4, 6, cyclin A, B, D E, cdc2, and cdc25, and by upregulating p21, p27, and p53, leading to apoptosis. As cell cycle arrest-mediated apoptosis is also an attractive drug target for cancer therapy, it is therefore imperative to explore DET and IDET for their potential to target cell cycle regulatory proteins in multiple in vitro and in vivo cancer models; this would make it possible to obtain additional evidence to support the development of these compounds into effective chemotherapeutic drugs. The mechanisms of DET and IDET-induced cell cycle arrest at multiple checkpoints in different cancer cell lines are summarized in Figure 2.

### 3.2. Extrinsic Apoptosis Pathway

Extrinsic apoptosis is one of the key apoptosis mechanisms triggered by death receptor activation [42]. Death receptors, or transmembrane protein receptors, are activated upon ligand binding on their extracellular cysteine-rich ligand binding domains. Death receptors have an intracellular death domain of ~80 amino acids that transmits death signals from the extracellular cysteine-rich ligand binding domain to intracellular signaling proteins [42,65,66]. The crucial death receptors include tumor necrosis factor receptor-1 (TNFR1), tumor necrosis factor receptor-related apoptosis-inducing ligand (TRAIL), fibroblast-associated antigen (Fas), DR3, DR4, DR5, and DR6 [65,66]. The activation of death receptors, especially Fas, DR4, and DR5, triggers the formation of death-inducing signaling complex (DISC) by interacting with the Fas-associated death domain (FADD) and procaspase-8 in the cell. Within the death effector domain of FADD, procaspase-8 is activated by auto-proteolysis and detached from DISC to induce apoptosis signaling either by type 1 extrinsic apoptosis pathway by directly activating downstream effector caspase-3, or by type 2 extrinsic apoptosis pathway through the truncation of Bid, a pro-apoptotic member of Bcl-2 proteins family [42,65,67].

**Figure 3:**
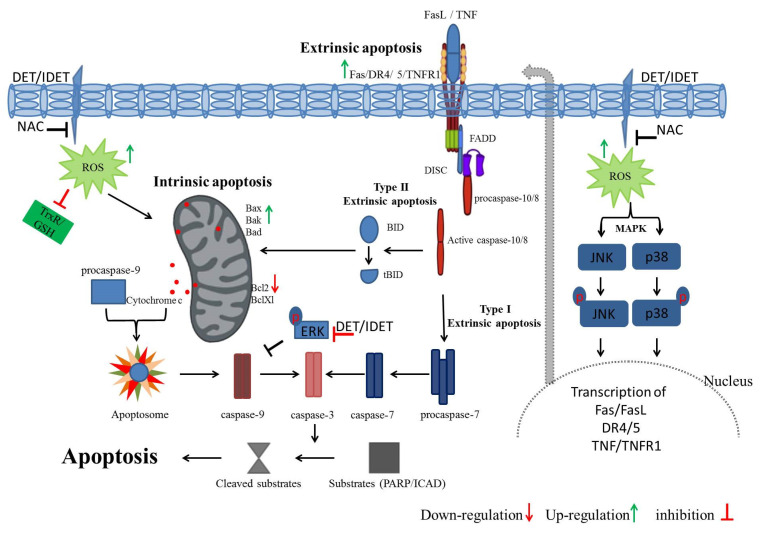
A schematic representation of DET/IDET-induced intrinsic and extrinsic apoptosis by ROS generation. DET/IDET induces ROS generation. Elevated ROS level activates JNK and p38 MAPK by phosphorylation. Activated p38 and JNK trigger the transcription of apoptosis-related genes, such as Fas/FasL, DR4/5, and TNF/TNFR1. Therefore, DET/IDET triggers the activation of extrinsic apoptosis and activates caspase-8 which, in turn, induces either tBid formation or downstream effector caspase-3 activation. Moreover, DET/IDET reduces thioredoxin reductase activity and intracellular level of glutathione, and induces intrinsic apoptosis by dissipating mitochondrial membrane potential and by modulating the expression of Bcl-2 family proteins, which results in caspase-3 activation. Caspase-3 then executes apoptotic cell death by cleaving PARP and activating ICAD.

DET-induced extrinsic apoptosis has been explored in multiple cancer cell lines, including cervical cancer, colorectal cancer, lung cancer, leukemia, and nasopharyngeal cancer. In nasopharyngeal carcinoma CNE cells, both intrinsic and extrinsic apoptosis are induced by DET. DET increased the expression of FasL, active caspase-10, -8, and -3, and Bid truncation at a high concentration (46.5 µM). Truncated Bid caused the release of cytochrome c by modulating Bcl-2 family proteins. Both type I and II extrinsic apoptotic pathways amalgamate at the caspase-3 and induce Poly(ADP-ribose) polymerase (PARP) cleavage, resulting in apoptosis [32]. In the lung adenocarcinoma A549 cell line, DET (12.28 μg/mL; 48 h) induced extrinsic apoptosis through the activation of procaspase-8 [37]. In murine mammary adenocarcinoma TS/A cells, DET (2 mg/mL) induced the upregulation of TNF-R1 protein and the activation of downstream caspase-8 through TNF/TNFR-mediated activation, rather than increasing the expression of Fas and FasL proteins [33]. In cervical cancer SiHa cells, DET (4.14 μg/mL; 48 h) induced extrinsic apoptosis through the activation of caspase-8 and downstream executioner caspase-7 [30]. In HCT 116, K562, KB, and T47D, DET (7.46, 4.02, 3.35, and 1.86 µg/mL, respectively) induced extrinsic apoptosis through the activation of caspase-8 and -7 and the upregulation of DR4, DR5, FasL, and TNF at the mRNA level. The activation of caspase-8 was cell type-specific [64]. Targeting DRs to induce extrinsic apoptosis in cancer cells has been considered as a promising approach to kill cancer cells. Collective lines of evidence suggest that DET holds the potential to induce extrinsic apoptosis by upregulating Fas/FasL, TNF/TNF-R1, DR4/5, active caspase-10, -8, -7, -3, and tBid formation in various cancer cells, which needs to be explored further using in vivo cancer models. The underlying mechanism of DET-induced extrinsic apoptosis pathway is shown in Figure 3.

### 3.3. Mitochondria-Mediated/Intrinsic Apoptosis

Intrinsic apoptosis, or mitochondrial-mediated apoptosis, is considered a potential therapeutic approach to treat cancers. Intrinsic apoptosis, unlike extrinsic apoptosis, is initiated by a diverse array of non-receptor-mediated extracellular and intracellular stimuli, including depletion of growth factors, oxidative stress, chemotherapeutic agents, DNA damage, hypoxia, and irradiation [40]. Key mediators of mitochondria-mediated/intrinsic apoptosis are B-cell lymphoma 2 (Bcl-2) family proteins. Modulation of Bcl-2 family proteins dissipates mitochondrial membrane potential (ΔΨm) and opens mitochondrial permeability transition pores, resulting in the release of pro-apoptotic proteins, including apoptosis inducing factor (AIF), cytochrome c, caspase-activated DNase (CAD), endonuclease G (Endo G), and second mitochondrial activator of caspases (Smac/DIABLO) from the mitochondrial inter-membrane space into cytosol [42,65,67]. In cytosol, caspase-9 activation is triggered through the interaction of cytochrome c with apoptosis protease activating factor-1 (Apaf-1), which subsequently activates caspase-3, leading to the cleavage of substrates e.g., the inhibitor of caspase-activated DNase (ICAD) and poly (ADP-ribose) polymerase (PARP), nucleosomal DNA fragmentation, and apoptosis [68,69,70].

DET has been demonstrated to induce intrinsic apoptosis in many types of cancer cell lines, including human osteosarcoma, pancreatic cancer, cervical cancer, breast cancer, lung cancer, nasopharyngeal cancer, colorectal cancer, and liver cancer cell lines through Bcl-2 family protein modulation. DET dose-dependently induces apoptosis through increased levels of pro-apoptosis Bax and decreased levels of anti-apoptosis Bcl-2, with the resultant activation of caspase-9, caspase-3, cytochrome c release, and PARP cleavage in human osteosarcoma cell lines, MG-63 and U2OS (4, 8, 16, and 32 µM) [71], and human pancreatic cancer cell lines, BxPC-3 (30 and 50 µM) and CFPAC-1 (40 and 60 µM) [72]. Similarly, Chim-Kei Chan et al. has reported that DET (0.75, 1.5, and 3.0 µg/mL) induced a concentration-dependent upregulation of cleaved caspase-3 and cleaved PARP protein expression in HCT116 cells [34]. In addition, DET (3 μg/mL; 6, 12 and 24 h) has been found to induce intrinsic apoptosis through the upregulation of Bax and B-cell lymphoma 2 antagonist/killer (BAK), with a concomitant decrease in Bcl-2 expression resulting in the dissipation of ΔΨm and the activation of caspase-9 and -3, as well as proteolytic degradation of PARP [73]. XIAP and Survivin, belonging to the inhibitor of apoptosis proteins (IAPs) family members, inhibit caspases and reduce apoptosis [74,75,76]. The second mitochondria-derived activator of caspase (Smac) and high temperature requirement protein A2 (HTRA2) are apoptogenic molecules that neutralize the effect of XIAP and Survivin, and promote caspase activation. DET downregulated Survivin and XIAP expression and activated caspase-9 and -3, resulting in intrinsic apoptosis in HCT116 cells [73]. DET (10 µM) induced intrinsic apoptosis in HeLa cells by activating caspase-9 and -3 and PARP cleavage [63]. It has been shown that DET (30 and 50 µM) induced intrinsic apoptosis in HepG2 cells by modulating Bax and Bcl-2, and by activating caspase-9 and -3 and PARP cleavage [36]. In a mouse mammary adenocarcinoma TS/A cell line, DET (2 mg/mL) induced apoptosis by targeting caspase-9, and -3 and by caspase-6-mediated specific cleavage of lamin and PARP at 36–48 h post-DET treatment [33]. The anti-cancer activity of DET has been reported in CNE cells, where DET treatment (up to 46.5 µM) induced a loss of ΔΨm from 3.7% to 48.4%. DET modulated the ratio of pro-apoptosis and anti-apoptosis proteins. DET (11.6, 23.2 and 46.5 µM) upregulated the expression of Bax, Bad, Bok, Bmf, and PUMA, downregulated the expression of Bcl-2 and Bcl-xL proteins, increased the expression of activated caspase-12, -9, -7, -3, and increased cytochrome release and PARP cleavage in a concentration-dependent manner [32]. In UL cells, DET (5, 10, and 25 µM) has been shown to upregulate Bax and caspase-3, and downregulate Bcl-2 both at the protein and the mRNA level, leading to an intrinsic apoptosis pathway [61]. DET (4.14 μg/mL; 48 h) downregulated Bcl-2 and Bcl-X_L_ and upregulated Bax at mRNA, thus altered the Bax/Bcl-2 ratio at the mRNA level with the activation of caspase-9, -7, and -3, released cytochrome c in cytosol, and induced PARP cleavage in cervical carcinoma SiHa cells [30]. Consistent with DET, IDET (10 µM) dissipated ΔΨm in MDA-MB-231 cells, and downregulated the expression of Bcl-xL and Bcl-2, and enhanced caspase-9, -7, and PARP cleavage at 1–5 µM [23]. All in all, it appears that DET and IDET hold the promise to induce intrinsic apoptosis by modulating Bcl-2 family proteins. As Bcl-2 family proteins are attractive therapeutic targets for cancer therapy, it is therefore imperative to explore these compounds using in vivo cancer models to assess their anti-cancer therapeutic potential. The mechanisms of DET and IDET-induced intrinsic apoptosis in cancer cells are summarized in Figure 3.

### 3.4. ROS-Mediated Apoptosis

A stable redox potential state is required to carry out normal cellular functions. Reactive oxygen species (ROS) or oxygen-containing reactive chemical entities are involved in various cellular processes, e.g., cell proliferation, cell survival, differentiation, enzyme activity, and gene expression [77,78]. Several studies have reported that there are higher levels of oxidative stress in cancer cells compared to normal cells due to their hypermetabolic state, which also disrupts cell death signaling, and causes drug resistance. Higher levels of oxidative stress enhance cancer cell survival, proliferation, angiogenesis, and metastasis [78,79]. Despite its tumorigenesis effect, this altered biochemical behavior of cancer cells can be exploited to yield therapeutic benefits. Cellular organelles and macromolecules, such as DNA, lipids, and proteins, under excessive oxidative stress experience oxidative damage, leading to cell death. Several in vitro and in vivo studies have shown that ROS-generating phytochemicals could pose oxidative damage, therefore selectively cause apoptosis of cancer cells by ROS-mediated mechanisms [78,79,80].

DET has been reported to induce apoptosis in hepatocellular carcinoma (HepG2), pancreatic cancer (BxPC-3, CFPAC-1, and PANC-1), murine mammary adenocarcinoma (TS/A), and cervical carcinoma (SiHa) cell lines by increasing ROS through various mechanisms. It has been reported that DET induced ROS-mediated apoptosis in HepG2 cells in a time- and concentration-dependent manner. DET (30 and 50 µM) reduced thioredoxin reductase (TrxR) activity, disrupted ΔΨm, depleted intracellular levels of glutathione (GSH), enhanced DNA fragmentation, and decreased the nuclear translocation of NF-κB through ROS production, since pretreatment with N-acetyl-L-cysteine (3 mM), an ROS scavenger, reversed the effect of DET [36]. DET increased the intracellular ROS level and induced ΔΨm in osteosarcoma MG-63 cells (DET at 16 μM) and U2OS cells (DET at 32 μM). The maximum ROS level was observed after 3 h of DET incubation in both cell lines [71]. In BxPC-3 and CFPAC-1 cells, ROS generation was induced as early as 0.5 h, and reached its maximum level at 2 and 3 h of DET incubation (50 and 60 µM, respectively). DET dissipated ΔΨm, modulated the GSH/GSSG ratio, and depleted Trx activity in BxPC-3 cells (DET at 30 and 50 µM) and CFPAC-1 cells (DET at 40 and 60 µM) [72]. DET (2 mg/mL) induced c-Jun N-terminal kinase (JNK) activation through ROS generation, causing cell death in TS/A cells. The DET-induced expression of c-Jun, p21, and the activation of JNK and p21 was reversed with NAC pretreatment, indicating the involvement of oxidative stress in DET-induced apoptosis [33]. DET (0.75, 1.5 and 3 μg/mL) also dissipated ΔΨm and induced oxidative stress in HCT116 cells. DET induced ROS-mediated autophagy and apoptosis via MAKP and PI3K/AKT/mTOR pathways in HCT116 cells [73]. In a very recent study, Biljana Cvetanova et al. reported the oxidative stress-mediated anti-proliferative and pro-apoptotic activities of DET, as well as of its synthetic derivative DETD-35, in A375LM5IF4g/Luc melanoma cells. They found that DET (6 µM) and DETD-35 (3 µM) elevated intracellular ROS levels by 68% and 168%, respectively, and depleted the GSH level. DET and DETD-35 induced mitochondrial superoxide generation in these cells, an indicator of excessive oxidative stress. DET-induced anti-proliferative and pro-apoptotic activities were reversed with the supplementation of GSH (5 µM), which suppressed ROS levels and mitochondrial superoxide generation. Furthermore, DET was found to decrease the basal oxygen consumption rate of A375LM5IF4g/Luc cells by interfering with mitochondrial bioenergetics and inhibiting mitochondrial oxidative phosphorylation [81]. Similarly, in CNE1 cells, IDET induced ROS generation in a concentration- and time-dependent manner. The anti-tumor activity of IDET (4 and 8 µM) was also found to be mediated by ROS generation, DNA fragmentation, decreasing mitochondrial cardiolipin resulting in ΔΨm, G2/M phase arrest, and upregulation of anti-tumor cytokines, including IL-12a and interferon (INF)-α, as well as INF-β. The reversal of all of these IDET-induced events was observed with the supplementation of NAC, except for INF-α and INF-β, indicating that IDET targeted both ROS-dependent and ROS-independent pathways [31]. These findings indicate that DET/IDET/DETD-35 hold the promise to induce ROS-mediated apoptosis by posing oxidative damage to cellular organelles via several mechanisms, including increased DNA fragmentation, increased mitochondrial superoxide generation, reduced ΔΨm, decreased mitochondrial cardiolipin, decreased basal oxygen consumption rate, reduced glutathione levels, and reduced thioredoxin reductase activity, as well as by targeting multiple signaling cascades, including decreased nuclear translocation of NF-κB, increased c-Jun N-terminal kinase (JNK) activation, increased c-Jun, p21 expression, activation of MAKP and PI3K/AKT/mTOR pathways, and upregulation of anti-tumor cytokines, i.e., IL-12a and interferon (INF)-α and INF-β. All of these ROS-mediated events lead to cancer cell death. It is imperative to further explore the anti-cancer therapeutic potential of these compounds using in vivo tumor models. The mechanisms of DET and IDET-induced ROS-mediated apoptosis are summarized in Figure 3.

## 4. Effect of DET and IDET on Multiple Pathways Involved in Cancer Progression

The pathogenesis and progression of cancer involves aberrant regulation of signaling pathways, including MAPK, Wnt/β-catenin, PI3K/AKT/mTOR, STAT3, and NF-κB pathways [82,83,84,85,86,87,88,89]. DET and IDET have been found to target these signaling pathways in several types of cancer cells.

### 4.1. Mitogen-Activated Protein Kinases (MAPKs) Pathway

Mitogen-activated protein kinases (MAPKs), including extracellular signal-regulated kinase-1 and 2 (ERK1/2), c-Jun N-terminal kinase (JNK), stress activated protein kinase (SAPK), and p38, are ubiquitous serine/threonine protein kinases, which have been implicated in many cellular processes, including tumor initiation, cell proliferation, differentiation, drug resistance, and apoptosis. The activation of ERK1/2 MAPKs promotes tumor cell proliferation, progression, and drug resistance, whereas JNK and p38 MAPK activation generally stimulates apoptosis. It has been shown in various studies that MAPKs are deregulated in many types of human cancers, and targeting these pathways are considered as an ideal approach for anti-cancer drug development. In A549 cells, DET (12.28 µg/mL; 48 h) did not alter total protein expression of ERK1/2, JNK, and p38, but inhibited the phosphorylation of ERK1/2 and enhanced p-p38 and p-JNK expression [90]. It is well established that ERK-MAPK inactivates caspase-9 by phosphorylation at Thr125 residue. The inhibition of ERK1/2 activation results in increased caspase-9 activation, leading to apoptotic cell death. In SiHa cells, the total ERK protein level was not changed in DET-treated cells (4.14 μg/mL; 48 h), while DET repressed ERK1/2 activation by inhibiting its phosphorylation. Consistent with this notation, DET inhibited ERK1/2 activation and enhanced caspase-9 activity, leading to apoptosis [30]. Although ERK1/2 promotes cell proliferation, there is growing evidence that anti-cancer drugs could prolong ERK1/2 activation and endorse apoptosis in tumor cells. For example, taxol, an anti-cancer agent, induced apoptosis through ERK1/2 activation in MCF7 cells [91]. Hispolon, a natural compound from a fungus *Phellinus linteus*, induced apoptosis by stimulating ERK1/2, p38, MAPK, and JNK1/2 in human nasopharyngeal and hepatocellular cancer cells [92,93]. In agreement with this notion, DET (3 μg/mL) induced the activation of ERK, p38, and JNK at 12 h post-treatment in HCT116 cells. Pre-treatment with p38-specific inhibitor (SB202190), JNK-specific inhibitor (SP600125), and ERK-specific inhibitor (UO126) significantly suppressed DET-induced activation of caspase-3 and PARP, indicating that DET-induced apoptosis was performed via MAPK pathways. Furthermore, pre-treatment with NAC reversed the DET-induced MAPK-mediated apoptosis, suggesting that DET induced MAPK-mediated apoptosis via ROS generation in HCT116 cells [73]. Moreover, a study of DET-induced cell death in CNE cancer cells revealed that DET (11.6–46.5 µM) induced the phosphorylation of JNK, AKT, and ERK. The expression levels of the total forms of AKT, ERK, JNK, and p38 remained unchanged at 6 h, with no phosphorylated p38 being detected. These findings suggest the involvement of JNK, AKT, and ERK in DET-mediated cell death [32]. In HCT 116, K562, KB, and T47D cancer cell lines, DET (7.46, 4.02, 3.35, and 1.86 µg/mL, respectively) attenuated the phosphorylation levels of ERK1/2, and increased the phosphorylation levels of SAPK/JNK and p38 MAPK without changing the protein expression level of ERK1/2, SAPK/JNK, or p38 MAPK [64]. DET (4.14 μg/mL) increased the phosphorylation of JNK and p38 without affecting total JNK and p38 protein levels in SiHa cells [30]. It has also been shown that JNK1/2 inhibition reduces liver damage and lethality in lipopolysaccharide/D-galactosamine- (LPS/D-GalN) challenged mice. Pre- and post-treatment with DET (10 mg/kg) inhibited the activation of JNK1/2 induced by LPS/D-GalN in ICR mice. Furthermore, the ratios of phospho-JNK1/JNK1 and phospho-JNK2/JNK2 were decreased by DET treatment, while silymarin (10 mg/kg), a hepatoprotective drug, had no effect on JNK1/2 phosphorylation. These findings indicate that DET induces MAPK-mediated cell death in different cancer cells [94]. The mechanisms by which DET targets MAPK pathway are shown in Figure 3.

### 4.2. Wnt/β-Catenin Signaling Pathway

**Figure 4:**
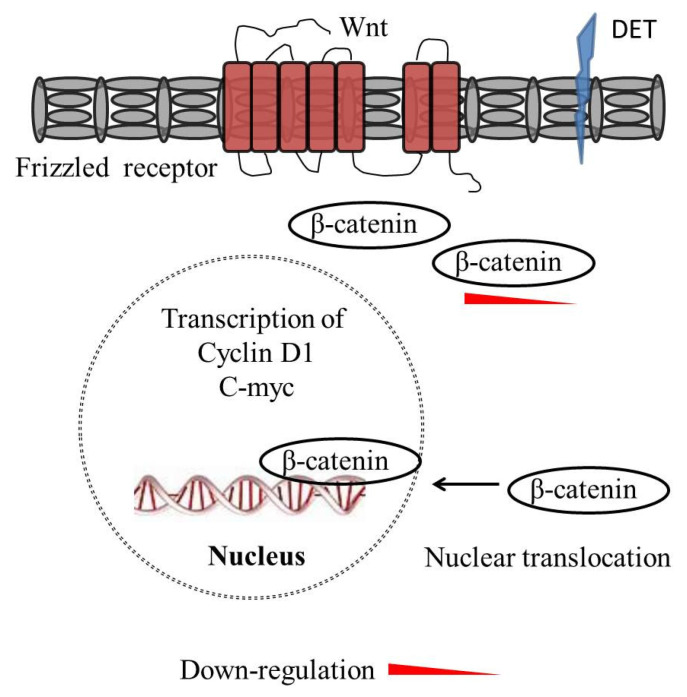
DET inhibits Wingless-Integrated/β-catenin (Wnt/β-catenin) signaling in cancer cells. The Wnt/β-catenin signaling pathway is involved in the regulation of various biological processes including cell division, migration, and fate determination during embryonic development. Over-expressed β-catenin regulates the transcription of oncogenes, including cyclin D1 and c-myc. DET downregulated the expression of β-catenin, cyclin D1, and c-myc.

The Wingless-Integrated/β-catenin (Wnt/β-catenin) signaling pathway is involved in the regulation of various biological processes, including cell division, migration, and fate determination during embryonic development. The Wnt pathway is either beta-catenin-independent (non-canonical) or beta-catenin-dependent (canonical). The Wnt/β-catenin pathway is composed of a family of ligands, receptors, and co-receptors, which are associated with signal transduction across the cell, and act as a regulator of cellular functions related to cancer initiation and progression. Over-expressed β-catenin regulates the transcription of oncogenes, including cyclin D1 and c-myc, in the nucleus. In HCT 116, K562, KB, and T47D cancer cell lines, DET inhibited the expression of β-catenin, cyclin D1, and c-myc at 7.46, 4.02, 3.35, and 1.86 µg/mL, respectively. These findings demonstrate that DET inhibits Wnt/β-catenin signaling in cancer cells [64]. The mechanisms by which DET inhibits Wnt/β-catenin signaling are shown in Figure 4.

### 4.3. STAT3 Signaling Pathway

Signal transducers and activators of transcription 3 (STAT3) is a member of the STAT protein family serving as a cytoplasmic transcription factor. STAT3 is perversely activated in multiple types of cancers, including breast, liver, lung, ovarian, and prostate cancers, as well as lymphoma [95]. STAT3 is activated by phosphorylation with tyrosine-705 or serine-727 amino acid residues. The activation of STAT3 is mediated by growth factor receptors, cytoplasmic kinases e.g., Janus activated kinases, cytokines e.g., interleukin6 (IL-6), Src family kinases, and ABL family kinases [96,97,98]. In total, two phosphorylated STAT3s in the form of dimers are translocated into the nucleus, and then bind to specific DNA sequences in the promoter region of their target genes, which are involved in the regulation of cell proliferation, differentiation, metastasis, angiogenesis, apoptosis, and immune response [98,99]. p53 inhibits the constitutive activation of STAT3 (Tyr705) due to the correlation between wild type p53 expression and STAT3 phosphorylation at Tyr705 [96]. In SiHa cells, DET (4.14 μg/mL) increased p53 expression and suppressed the phosphorylation of STAT3 at Tyr705. The inhibition of STAT3 activation caused downregulation of STAT3-regulated gene products, including Bcl-2 and Bcl-xL [30].

**Figure 5:**
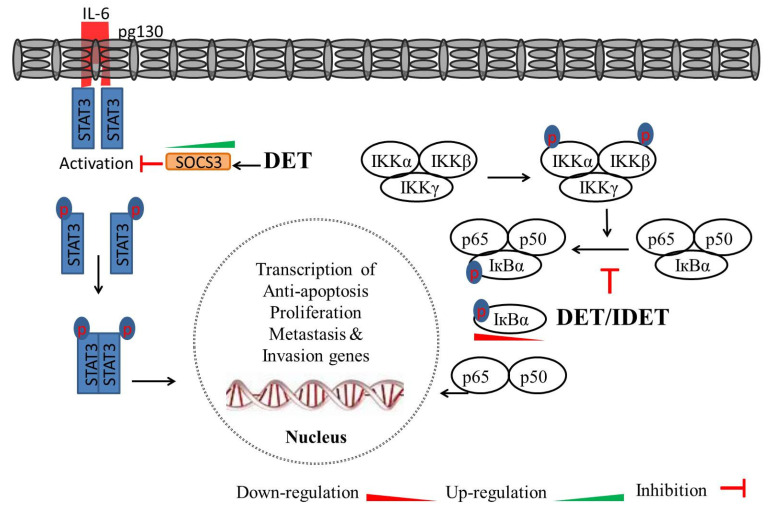
A schematic model of DET/IDET-induced STAT3 and NF-κB inhibition. DET upregulates the expression of suppressor of cytokine signaling 3 (SOCS3), which is a major component for the negative regulation of IL-6 signaling cascade, and inhibits STAT3 activation by reducing phosphorylation at tyrosine 705. DET/IDET inhibits NF-κB activation induced by various inflammatory stimuli (TNF, LPS, and IL-1β), which activate NF-κB through different pathways. The inhibition of NF-κB activation was associated with decreased phosphorylation and degradation of IκB-α, as well as upstream activation of IKK-α/-β and IKK kinase activities. DET/IDET downregulates the expression of NF-κB- and STAT3-regulated gene products involved in anti-apoptosis, cell proliferation, metastasis, and invasion.

It has been established that, in macrophages, lipopolysaccharides (LPS) activate STAT3, leading to the production of proinflammatory cytokines, including IL-6 [94]. The activation of STAT3 by IL-6/IL-6R is critical for inflammation. Chi-Chang Huang et al. reported that, in male ICR mice liver insulted with lipopolysaccharide/D-galactosamine (LPS/D-GalN), pre- and post-treatment with DET (10 mg/kg) increased STAT3 accumulation, inhibited STAT3 phosphorylation, and decreased the ratio of p-STAT3/STAT3. The effect of DET was comparable to that of the hepatoprotective drug silymarin (10 mg/kg). Furthermore, in LPS/D-GalN-insulted mice, the protein level of the suppressor of cytokine signaling 3 (SOCS3), a negative regulator of IL-6 signaling, was decreased. DET (10 mg/kg) and silymarin (10 mg/kg) treatment increased SOCS3 expression, suggesting that DET protected against acute liver inflammation through the IL-6/STAT3 pathway [94]. The mechanisms underlying the effect of DET on STAT3 pathways are shown in Figure 5.

### 4.4. NF-κB Signaling Pathway

Nuclear factor-kappa B (NF-κB), a family of transcription factors, consists of NF-κB1 (p105/p50), NF-κB2 (p100/p52), RelA (p65), RelB, and c-Rel [100]. The constitutive activation of NF-κB mediates transactivation of several essential genes involved in tumor cell proliferation, invasion, metastasis, and angiogenesis [101]. Under normal physiological conditions, inhibitors of κBs (IκBs) arrest the translocation of NF-κB from the cytoplasm to the nucleus. Several stimuli, such as microbial pathogens, carcinogens, endotoxins, free radicals, interleukin 1 (IL-1), interleukin 6 (IL-6), lipopolysaccharide (LPS), tumor-necrosis factor (TNF), tumor promoter, and radiation (UV-light, X-rays, γ-rays), trigger the activation of NF-κB [4]. IκBs are phosphorylated by IκB kinases (IKK), which results in the activation of NF-κB. IKK is composed of one regulatory subunit IKK-γ and two catalytic subunits IKK-α and IKK-β, which are involved in ubiquitination or proteasomal degradation of IκBs [4,102]. The p50/p65 complex, the most common active form of NF-κB, is released after the phosphorylation of IκB-α by IKK, and is transported into the nucleus, where it initiates the transcription of specific oncogenes and anti-apoptotic genes (survivin, Bcl-2 family protein, IAP) [103]. The effect of DET on NF-κB activity has previously been investigated in HepG2 liver cancer cells. DET (30 and 50 µM) inhibited both constitutive activation of NF-κB and TNF-α- and the gemcitabine-induced activation of NF-κB. DET (30 µM) produced the same inhibitory effect as that of IKK-16, an NF-κB inhibitor. The inhibition of both constitutive and inducible activation of NF-κB by DET was found to be associated with decreased phosphorylation of IκB-α [36]. Consistent with this notion, DET (30 and 50 μM) has been shown to inhibit NF-κB activity through the downregulation of p50 and p65 expression in BxPC-3 cells. DET upregulated IκB-α, downregulated p-IκB-α, and inhibited the translocation of NF-κB from the cytoplasm into the nucleus. DET inhibited the translocation of NF-κB-p65 in the same way as IKK-16. DET (30 μM) inhibited TNF-α- (50 ng) and gemcitabine-induced (50 μM) NF-κB activation with a concomitant regulation of related downstream gene products of NF-κB, including Ki-67, PCNA, and E-cadherin [72]. DET (12.28 µg/mL) has been shown to induce the inhibition of NF-κB activation at the transcriptional level by reducing the gene expression of NF-κB and IκB in A549 cells. Many studies have shown that constitutive or inducible NF-κB activation stimulates genes involved in invasion, such as MMP-9/-2, uPA, and uPAR. DET inhibited MMP-9/-2, uPA, and uPAR, resulting in metastasis inhibition [90]. In TS/A cells, DET (1–5 μg/mL) inhibited TNF-α-induced NF-κB activity, and downregulated NF-κB-regulated gene products involved in invasion, including MMP-9/-2, resulting in the suppression of migration and invasion of TS/A cells in vitro and in vivo. The DET-mediated blockage of NF-κB-p65 was associated with the formation of hydrogen bonds between the carbonyl group at position 16 of DET and the Lys122 residue of NF-κB-p65 [33].

IDET (10 µM) inhibited the constitutive activation of NF-κB in human myeloma MM.1S and U266 cells, as well as head and neck squamous cell carcinoma SCC4 and LICR-LON-HN5 cells, which are known to express constitutive NF-κB activation. In human chronic myeloid leukemia KBM-5 cells, DET and IDET inhibited TNF-α-induced NF-κB activity, and maximum inhibition was observed between 10 to 50 µM. IDET (10 µM) suppressed TNF-α, PMA-, lipopolysaccharide-, and IL-1β- induced NF-κB activity in KBM cells, indicating that the effect of IDET on the inhibition of NF-κB activation shares common targets among all these proinflammatory agents. IDET inhibited NF-κB activity in MCF-7 cells, H1299 cells, and HL60 cells, suggesting the broad-spectrum effect of IDET on inhibiting NF-κB activity. The effect of IDET on NF-κB activity was mediated by the inhibition of IKK activation, the inhibition of IκB-α phosphorylation and degradation, and the inhibition of p65 phosphorylation and nuclear translocation in a variety of cell lines. NF-κB regulates genes involved in cell proliferation e.g., COX-2, cyclin D1, and c-Myc; invasion e.g., MMP-9 and ICAM-1, as well as anti-apoptosis e.g., IAP1, IAP2, Bcl-2, Bcl-xL, Bfl-1/A1, TRAF1, FLIP, and Survivin. IDET (2 µM) reduced the expression of TNF-α -induced COX-2, cyclin D1, c-Myc, MMP-9, ICAM-1, IAP1, IAP2, Bcl-2, Bcl-xL, Bfl-1/A1, TRAF1, FLIP, and Survivin in KBM-5 cells [17]. In MDA-MB-231, IDET (10 µM; 6 h) inhibited the okadaic acid (OA)-induced p65 nuclear translocation. Furthermore, IDET has been shown to exhibit superior NF-κB inhibition and pro-apoptotic activities over DET in MDA-MB-231 cells. The affinity of p65 binding with IDET was sturdier than that of DET, as indicated by binding energies (−6.67 vs. −6.51 kcal/mol), the dissociation constant (K_i_ = 12.86 vs. 16.99 µM), and hydrogen bonds (three vs. two), suggesting that IDET has a stronger p65 inhibitory effect than DET does [23]. The mechanism which deregulates NF-κB activity might be different in different cancers. Collective data suggest that DET and IDET hold the potential to inhibit both constitutive and inducible (induced by gemcitabine, TNF-α, PMA-, lipopolysaccharide-, IL-1β-, and okadaic acid) NF-κB activation in various cancer cells, therefore illustrating the broad-spectrum of the tumor inhibition activity of these compounds. In general, DET and IDET induce NF-κB inhibition by the inhibition of IKK activation, the upregulation of IκB-α, the inhibition of IκB-α phosphorylation and degradation, and the inhibition of p65 phosphorylation and nuclear translocation. The inhibition of nuclear translocation of p65 prevents the transcription of NF-κB-regulated gene products, including Ki-67, PCNA, E-cadherin, MMP-9/-2, uPA, uPAR, COX-2, cyclin D1, c-Myc, MMP-9, ICAM-1, IAP1, IAP2, Bcl-2, Bcl-xL, Bfl-1/A1, TRAF1, FLIP, and Survivin. Taken together, DET and IDET inhibit NF-κB activation and induce apoptosis by inhibiting cell proliferation, anti-apoptosis, and invasion in various cancer cells. Further studies should be focused on assessing the anti-cancer efficacy of these compounds against these signaling pathways in vivo. The mechanisms by which DET and IDET inhibit NF-κB activation and NF-κB-regulated gene expression are summarized in Figure 5.

### 4.5. PI3K/AKT/mTOR Pathway

**Figure 6:**
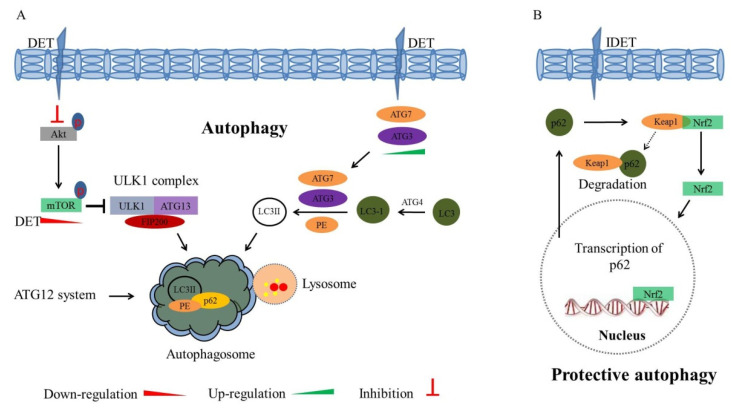
Schematic diagram illustrating DET-induced autophagy and IDET-induced protective autophagy. (**A**) DET-induced autophagy via the inhibition of PI3K/AKT/mTOR signaling. DET inhibits the phosphorylation of AKT and mTOR, which results in ULK1 complex formation. DET up-regulates the expression of ATG3, ATG5, and ATG7, and induces the conversion of LC3I to LC3II conversion. The ULK1, LC3II, and ATG12 systems form autophagosomes. (**B**) IDET-induced protective autophagy via Nrf2-p62-Keap1 feedback loop. Under normal physiological conditions, Nrf2 is transferred by keap1 to proteasome for degradation. Upon the induction of oxidative stress, Nrf2 is released from Nrf2-keap1 complex, and is translocated to the nucleus, where it activates the transcription of HO-1 and p62, which are downstream targets of Nrf2. Generally, p62 is downregulated during autophagy. IDET activates Nrf2 signaling and upregulates its downstream target p62. Upregulated p62 binds competitively with Keap1, and Nrf2 is released from the Nrf2-Keap1 complex, then is translocated to the nucleus, where it further stimulates the transcription of p62. Conclusively, IDET induces protective autophagy via the Nrf2-p62-Keap1 feedback loop mechanism.

Phosphatidylinositol-3-Kinase (PI3K)/AKT, belonging to the serine/threonine kinase protein family, play an important role in tumor cell proliferation, growth, survival, and apoptosis resistance. Phosphorylation of AKT at threonine 308 (Thr308) and serine 473 (Ser473) amino acid residues results in AKT activation, which further stimulates several transcription factors, thereby, leading to the increased transcription of anti-apoptotic and pro-survival genes. A downstream serine/threonine protein kinase, a mammalian target of rapamycin (mTOR), is also activated by p-AKT, which is an important regulator of cell growth, proliferation, and apoptosis. The activation of mTOR is associated with poor prognosis in cancer patients [104,105]. The inhibition of AKT and its associated downstream transcription factors are considered as a potential therapeutic strategy in cancers. In HCT116 cells, DET (3 μg/mL) suppressed pyruvate dehydrogenase kinase 1 (PDK1) and inhibited the phosphorylation of AKT at Ser473 and Thr308 residues in a time-dependent manner. Furthermore, DET markedly reduced the expression levels of the phosphorylated phosphatase and tensin homolog (PTEN), which is an inactive form of PTEN. The expression levels of both phosphorylated-mTOR and regulatory-associated protein of mTOR (RAPTOR) were significantly suppressed in DET-treated cells (12 and 24 h) [73]. The suppression of AKT induces apoptosis by activating pro-apoptotic factors, including Bad and caspase-9. In HCT116, K562, KB, and T47D cancer cells, DET (7.46, 4.02, 3.35, and 1.86 µg/mL, respectively) did not alter total AKT protein level, but significantly decreased the expression level of p-AKT and p-mTOR. The suppression of AKT leads to increased expression of caspase-9 and Bad. It has been established that the activation of mTOR inhibits autophagy, and the inhibition of mTOR induces autophagy in cancer cells [106]. DET was found to downregulate mTOR and induce autophagy-mediated cell death, indicating that DET inhibition of PI3K/Akt/mTOR signaling leads to autophagic cell death [64]. In addition, DET (4.14 μg/mL) decreased the phosphorylation of AKT and mTOR in SiHa cells [30], whereas DET upregulated the expression of the p-AKT in CNE, indicating that the effect of DET on AKT depends on the type of cancer cell [32]. The mechanisms by which DET affects the PI3K/AKT/mTOR pathway are summarized in Figure 6A.

## 5. Effect of DET and IDET on Autophagy Pathways

Autophagy is a highly dynamic catabolic process which is important for maintaining cellular homeostasis. It degrades misfolded proteins and damaged organelles, and eliminates harmful cellular components [107,108]. Under physiological conditions e.g., starvations, and pathological conditions e.g., infections, autophagy presents protective roles facilitating cell survival [107,108,109]. However, excessive autophagy can result in autophagic cell death. The induction of excessive autophagy or autophagic cell death has been regarded as an alternative therapeutic strategy for apoptosis-defective or apoptosis-resistant cancer cells. Several studies have shown that natural compounds exert autophagic cell death responses in apoptosis-defective cancer cells [110,111]. The intricate interplay between autophagy and apoptosis is currently being investigated for cancer treatment. It has been reported that the inhibition of protective autophagy induces apoptosis in cisplatin and 5-fluoroucil-treated cancer cells [109]. Other studies showed that the inhibition of Beclin, an autophagy gene, by Bcl-2 protein resulted in an inhibition of autophagy. Bcl-2 inhibition induced both apoptosis and autophagy [111]. Identification of autophagic modulators and characterization of intricate interplay between apoptosis and autophagy might lead to effective anti-cancer treatment strategies.

In HCT116 cells, DET induced the formation of reddish orange acidic vesicles, autophagosomes, which are doubled membrane-bound vesicles containing residual debris or digested material of cells [73]. An autophagy-related protein, LC3, is a hallmark of autophagy, and the conversion of LC3-I to LC3-II might occur by increased up-stream autophagosome formation, or through damage to downstream autophagosome-lysosomes fusion [112,113]. DET (3 µg/mL) increased the expression of autophagy-related proteins LC3A/B-II, ATG7, and ATG5 in a time-dependent manner, and decreased p62 expression at 24 h post-treatment. Pre-treatment with 3-MA, an autophagy inhibitor, reduced the DET-induced expression of LC3A/B-II and autophagy, which, in turn, reduced apoptosis induction. Furthermore, pre-treatment with an apoptosis inhibitor, z-VAD-FMK, significantly inhibited the expression of caspase-3, with a concomitant increase in LC3A/B-II proteins. An accumulation of bright red acidic vesicles was observed in z-VAD-FMK pre-incubated DET-insulted cells. Overall results suggest that the inhibition of autophagy promotes apoptosis, while the inhibition of apoptosis endorses autophagy. Moreover, the knockdown of LC3 which facilitates the formation of autophagosomes by siLC3 siRNA, partially reversed the DET (3 µg/mL)-induced cytotoxicity and expression of apoptosis markers, and cleaved PARP was reduced, indicating that the pro-apoptotic effect triggered by DET was interrupted in the absence of autophagy. These findings confirm that DET activates autophagy-dependent apoptosis in HCT116 cells [73]. It has also been shown that DET upregulated LC3II levels in MG-63 and U2OS cells. Chloroquine inhibits lysosomal degradation by neutralizing lysosomal pH. In DET-treated MG-63 cells (8 µM; 24 h), the LC3II level was increased with the addition of chloroquine. Moreover, mRFP-GFP-LC3 adenovirus transfection analysis further confirmed that levels of autophagosomes and autophagosome-lysosomes were increased in DET-treated osteosarcoma cells. Despite the presence of autophagy in DET-treated osteosarcoma cells, the apoptosis rate was not changed upon chloroquine pre-incubation, indicating that there might be some other mechanisms involved in DET-induced cell death [71]. The mechanism of DET-induced autophagy is summarized in Figure 6A.

It has been shown through confocal fluorescence microscopy analysis that low concentrations of IDET significantly induced autophagy flux in lung cancer H1299 and A549 cells expressing the mCherry-EGFP-LC3 reporter. IDET (0.4–3.2 µM) increased the expression levels of autophagy markers such as LC3-II, ATG3 and Beclin1. Furthermore, the autophagy flux was demolished by pretreatment with 3-MA. Interestingly, at lower concentrations of IDET (1.6 μM for H1299 and 3.2 μM for A549), 3-MA significantly enhanced the antitumor activity of IDET, suggesting that the repression of protective autophagy enhances the effect of IDET in inducing apoptosis and inhibiting growth of lung cancer cells. Besides autophagy, nuclear factor erytheroid-derived-2-like 2 (Nrf2) is another signaling molecule that provides adaptive protection against oxidative and proteotoxic stress in cells. Under normal physiological conditions, Nrf2 is transferred by keap1 to proteasome for degradation, while Nrf2 is released from the Nrf2-keap1 complex upon induction of oxidative stress, and is translocated to the nucleus, where it activates the transcription of downstream target genes related to autophagy, proliferation, and metastasis. However, the knockdown of Nrf2 by small interfering RNA (siRNA) rendered cancer cells more susceptible to chemotherapeutic agents. It has been demonstrated that IDET (3.2 µM; 24 h) significantly affected Nrf2 in A549 cells. Likewise, IDET (1.6 μM) induced Nrf2 activation and Nrf2 nuclear expression in H1299 cells. Furthermore, IDET significantly increased the expression levels of HO-1 and p62, which were downstream targets of Nrf2, and increased the expression levels of autophagy markers, including ATG3, Beclin1, and LC3-ll. In Nrf2-knockdown of H1299 and A549 cells, IDET-induced expression of p62, HO-1, ATG3, Beclin1, and LC3-ll was reduced, with a concomitant increase in anti-cancer activity of IDET. Interestingly, pretreatment with 3-MA failed to diminish the IDET-induced Nrf2 activation, suggesting that Nrf2 activation is required for IDET-induced autophagy. It is well established that p62 binds to ubiquitylated protein aggregates to deliver them to autophagosomes for degradation. Generally, p62 is downregulated during autophagy. Unexpectedly, p62 was upregulated in IDET-induced autophagy at both the mRNA and protein levels in H1299 and A549 cells. IDET activated Nrf2 signaling, and upregulated its downstream target p62. Upregulated p62 binds competitively with Keap1 and releases Nrf2 from the Nrf2-Keap1 complex, allowing Nrf2 translocation to the nucleus. The knockdown of p62 significantly blocked IDET-induced autophagy, and markedly enhanced anti-cancer effect of IDET to be similar to the effect of autophagy inhibition by 3-MA in EH1299 and A549 cells [114]. These findings suggest that IDET induces protective autophagy via an Nrf2-p62-Keap1 feedback loop, and the inhibition of autophagy enhances IDET-induced cytotoxicity in lung cancer cells as summarized in Figure 6B.

## 6. Effect of DET on Cancer Invasion and Metastasis

In addition to its effect on enhancing cell death, DET has been shown to inhibit cancer invasion and metastasis, which is a migration of cancer cells to surrounding tissues or to elsewhere in the body through circulation, respectively. Invasion and metastasis are sequential hallmark properties of malignant tumor cells. Degradation of the basement membrane and extracellular matrix (ECM) sets the initiation of cancer invasion and metastasis. Matrix metalloproteinases (MMPs) are involved in invasion and metastasis, and are inhibited by endogenous tissue inhibitor of matrix metalloproteases (TIMPs) e.g., TIMP-1 and TIMP-2 in normal cells. In cancer cells, the MMPs and TIMPs balance is disturbed, leading to invasion and metastasis. In A549 cells, DET (12.28 µg/mL; 48 h) has been shown to downregulate the expression of MMP-9 and -2, and upregulate the expression of TIMP2 without changing the expression of TIMP1 at mRNA level. Urokinase-type plasminogen activator receptor (uPAR), which is a GPI-anchored cell membrane receptor, controls urokinase (uPA) proteolytic activity on the cell surface. The binding of uPA to its receptor uPAR activates MMPs and triggers ECM degradation, leading to invasion and metastasis. DET inhibited mRNA expression of uPA and uPAR, and inhibited the metastatic ability of A549 cells by the downregulation of MMP-2, MMP-9, uPA, and uPAR, and the upregulation of TIMP2 at mRNA level [90]. In TS/A cells, DET (0.5–2 µg/mL) suppressed the constitutive and TNF-α-induced enzymatic activity of MMP-9 and -2. DET decreased MMP-9 protein expression [33]. It has also been shown that wound healing and cell migration activity were decreased in HCT 116, K562, KB, and T47D cancer cells treated with DET at 7.46, 4.02, 3.35, and 1.86 µg/mL, respectively. Furthermore, DET reduced constitutive mRNA expression of MMP-9/-2, uPA, and uPAR, with a substantial increase in TIMP-1/-2 mRNA levels in HCT 116, K562, KB, and T47D cancer cells [64].

## 7. DET-Derivatives (DETDs)

**Figure 7:**
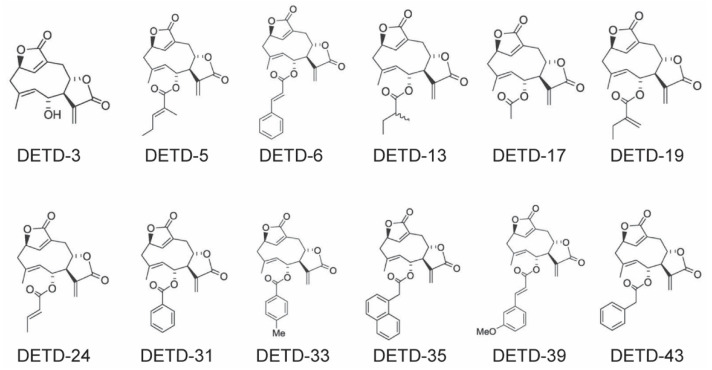
Chemical structures of DET derivatives.

Accumulative lines of scientific evidence confirm pro-apoptotic, anti-invasive, and anti-metastatic properties of DET. In order to develop this class of compounds into anti-cancer drugs, DET derivatives (DETDs) were synthesized and evaluated for their anti-cancer activities, including DETD-3, -5, -6, -13, 17, -19, -24, -31, -33, -35, -39, and -43. These compounds were developed through a semi-organic method, and tested against melanoma cells and normal melanocytes [115]. The chemical structures of DETDs are shown in Figure 7. It has been shown that DET and all synthesized DETDs showed different levels of toxicity against wild-type as well as mutant melanoma cell lines B16-F10, MeWo, A375, A2058, SK-MEL-2, and normal melanocytes. DETD-5 (IC_50_ values of 4.1–7.8 µM) showed comparable toxicities against melanoma cells, except wild-type MeWo and normal melanocytes, while DETD-13, -17, -19, and -24 did not show toxic effects against human melanoma cells. Among other DEDTs, DEDT-6 (2.5–6.0 µM), -33 (3.9–9.9 µM), and -39 (1.6–3.5 µM) inhibited melanoma cell proliferation with slight toxicity on normal melanocytes. DETD-35 (2.2–6.7 µM) demonstrated potent toxicity against melanoma cells, with little or no toxicity against normal melanocytes. These findings suggest that, among all DETDs, DET-35 represents the most favorable compound against cancer cells. Studies on DETD-35 showed that DETD-35 is three times more potent against cancers, and less or not at all potent against normal cells, compared to DET [116]. The mode of action of DETD-35 against cancers is same as that of DET. Several studies have shown that DETD-35 induces ROS-mediated apoptosis through multiple pathways, including Bcl-2 family protein modulation, dissipation of ΔΨm, cell cycle arrest at the G2/M phase, the inhibition of ERK phosphorylation, the induction of autophagy, and the inhibition of metastasis in various cancer cells, including melanoma and triple negative breast cancer cells [81,115,116,117]. Although DETD-35 has shown promising antitumor activity against melanoma, more comprehensive mechanistic research using in vivo and in vitro studies on different cancer cell models is required to evaluate the real potential of DETD-35 as an anti-cancer agent.

## 8. Safety Profile of DET/IDET/DETD-35

Natural compounds of plant origin have long been used to cure various diseases, including cancers. Due to structural diversity and broad-spectrum properties, these compounds have received enormous attention as alternative medicines for the treatment of cancers. The safety profiles of compounds are the key factor to be considered for the development of effective anti-cancer drugs. An ideal anti-cancer agent should be selective towards cancer cells, and nontoxic or minimally toxic to normal cells. Safety and efficacy of natural products should be thoroughly studied before adopting them as anti-cancer agents. In this review, we have summarized that DET induced cytotoxicity against human and murine tumor cells. The presence of both α-methylene-γ-lactone ring and α, β-unsaturated cyclopentenone is considered a bioactive functional group responsible for cytotoxicity of DET. DET (35 µg/mL) did not induce toxicity in peripheral blood lymphocytes isolated from healthy individuals, suggesting that DET specifically induced toxicity in cancer cells [37]. Miaoxian Su et al. showed that a major proportion of ethanolic extracts of *Elephantopus scaber* is composed of DET, which produces a weak effect on the proliferation of human normal skin fibroblast Hs68 cells with IC_50_ values of >500 g/mL, suggesting a differential cytotoxic effect of DET on cancer and normal cells [62]. Chim Kei Chan et al. reported that the IC_50_ values of DET and IDET were 0.73 µg/mL (2.12 µM) and 0.88 µg/mL (2.56 µM), respectively, in HCT116 cells. DET at 21.69 µg/mL (60.02 µM) was relatively less cytotoxic to the CCD841CoN normal cells compared to HCT116 cells, revealing a 30-fold difference in cytotoxicity [34]. In H1299 and A549 cells, IDET produced cytotoxicity at various concentrations, peaking at 51.2 μM at 24 and 48 h. Intriguingly, IDET showed markedly less cytotoxicity toward non-cancer lung epithelial HBE cells [114]. It has been shown that while IDET treatment (4–12 µM; 24 h) induced G2/M phase cell cycle arrest in CNE1 and SUNE1 cells, it did not induce cell cycle arrest at any phases in NP69 normal nasopharyngeal cells [31]. The prolongation of the life span is an ideal characteristic of potential anti-cancer drugs. It has been shown that DET (25 mg/kg, i.p.) increased the life span of Dalton’s ascitic tumor cells (DLA)-bearing mice, and displayed a significant antitumor effect in vivo. The life span-increasing property of DET was comparable to that of vincristine (1 mg/kg), a standard anti-cancer drug. DET, at higher doses (50 mg/kg, i.p and 100 mg/kg, i.p), exhibited anti-cancer activity with an increase in life span of animals, which is a reliable criterion for evaluating the promise of anti-cancer drug [25]. Using an Ehrlich’s ascites carcinoma (EAC)-tumor model in Swiss albino mice, treatment with DET (10 mg/kg) and 5-FU (25 mg/kg), a standard anti-cancer drug, significantly increased the mean survival time and percentage of the life span of mice compared to mice in control groups. In an EAC-solid tumor model, DET (25 mg/kg) prolonged the overall survival of EAC solid tumor-bearing mice, with similar tumor reduction capability as that of 5-FU [118]. It has been shown that the structural derivative of DET, DETD-35 (2.2–6.7 µM), shows little or no toxicity against normal melanocytes [116]. Therefore, DET/IDET represent a group of natural compounds which have a good safety profile and favorable pharmacological properties as anti-cancer agent.

## 9. Conclusions and Future Perspectives

In this review, we have summarized the scientific evidence on anti-cancer properties of DET, IDET, and its derivatives, including DETD-35, in various cancer cell lines. Collective data from different reports indicate that DET, IDET, and DETD-35 are promising candidates for developing anti-cancer agents. This notion is supported by the fact that (I) DET, IDET, and DETD-35 show broad-spectrum therapeutic potential toward various human cancers; (II) these compounds have been shown to induce cancer cell death by inducing cell cycle arrest, as well as intrinsic and extrinsic apoptosis in cancer cells; (III) these compounds target multiple signaling pathways (e.g., NF-κB, STAT3, ERK, and PI3k/AKT/mTOR) involved in the pathogenesis and progression of human cancers, as well as chemotherapeutic resistance; (IV) unlike other drugs that are severely toxic to normal cells, DET and IDET, as the major components of *Elephantopus scaber* and *Elephantopus carolinianus* (which have long been used in traditional medicines to cure various ailments), are considered to be safe chemotherapeutic compounds for the treatment of cancer. The aforementioned findings illustrate that DET, IDET, and DETD-35 may become potential compounds for anti-cancer drug development. However, additional preclinical and clinical developments are required to explore the entire spectrum of anti-cancer activity and pharmacological as well as toxicological properties of this class of compounds to endorse their clinical utility as anti-cancer agents.

## Data Availability

Not applicable.

## Sample Availability

Samples of the compounds are available from the authors.
